# The association between *Toxoplasma* infection and mortality: the NHANES epidemiologic follow-up study

**DOI:** 10.1186/s13071-022-05398-1

**Published:** 2022-08-06

**Authors:** Jiaofeng Huang, Jiaolong Zheng, Bang Liu, Lingling Lu, Haicong Wu, Su Lin, Dongliang Li

**Affiliations:** 1 Department of Hepatobiliary Disease, Fuzong Clinical Medical College of Fujian Medical University, Fuzhou, China; 2 Department of Hepatobiliary Disease, The 900th Hospital of Joint Logistics Support Force, Fuzhou, China; 3 Department of Hepatology, Hepatology Research Institute, The First Affiliated Hospital of Fujian Medical University, Fuzhou, China

**Keywords:** *Toxoplasma gondii*, Toxoplasmosis, Mortality, NHANES

## Abstract

**Background:**

*Toxoplasma gondii* has been reported to be associated with higher mortality in patients with schizophrenia. This study aimed to explore the relationship between *T. gondii* infection and 25-year mortality based on data from the Third National Health and Nutrition Examination Survey (NHANES III) database.

**Methods:**

Cases with serum *T. gondii* antibody test results were included in this study and the corresponding mortality dataset was obtained from the US National Center for Health Statistics (NCHS). Propensity score matching (PSM) was used to match age and sex between groups. The Cox proportional hazards model was used to evaluate the effect of *T. gondii* infection on mortality.

**Results:**

A total of 14,181 cases were included in the analysis, of which 3831 (27.0%) were seropositive for *T. gondii* antibody. The median follow-up time of the whole cohort was 22.5 (interquartile range 16.3, 24.5) years. A total of 5082 deaths were observed in this cohort, a mortality rate of 35.8%. All-cause mortality was significantly higher in the seropositive group than in the seronegative group (50.0% vs 30.6%, *P* < 0.001). Kaplan–Meier analysis showed a significant difference in the survival time between two groups before and after PSM. Multivariate analysis showed that *T. gondii* infection was independently associated with higher all-cause mortality after adjusting for potential confounders.

**Conclusions:**

*Toxoplasma gondii* infection is associated with higher mortality in general population.

**Graphical Abstract:**

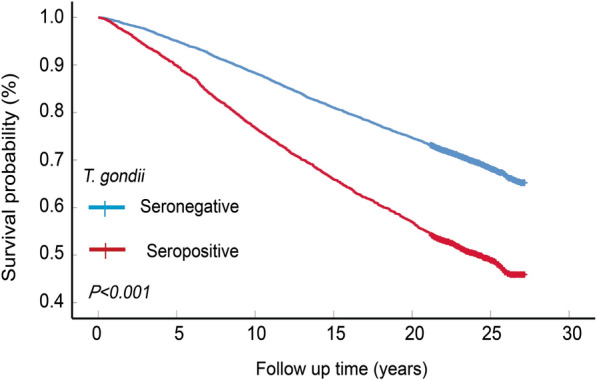

**Supplementary Information:**

The online version contains supplementary material available at 10.1186/s13071-022-05398-1.

## Background

Zoonoses continue to be a major public health concern [1]. *Toxoplasma gondii* is a common protozoan parasite that infects approximately 30% of the world’s population [2, 3]. In healthy individuals, *T. gondii* infection usually results in mild illness most of the time, but it has the potential to cause severe consequences. Toxoplasmosis is a common opportunistic infection in immunocompromised patients and results in high mortality in this patient population [2, 4, 5].

Toxoplasmosis has been linked to a number of diseases, including neuropsychiatric diseases, such as primary neuropathies, behavioral and psychiatric disorders [6, 7], as well as some related metabolic diseases, such as non-alcoholic fatty liver disease [8] and diabetes [9, 10]. *Toxoplasma gondii* infection is also associated with a worse prognosis of all-cause mortality in individuals with these diseases; for example, it has been shown to be associated with higher all-cause mortality rate in patients with schizophrenia [11, 12]. However, it remains unclear whether* T. gondii *infection actually affects the all-cause mortality rate in the general population.

The present study aimed to explore the relationship between *T. gondii* infection and 25-year mortality in the general population based on data retrieved from a nationally representative longitudinal database.

## Methods

### Study population

The study population was from the Third National Health and Nutrition Examination Survey (NHANES III) 1988–1994, which was a periodic survey conducted by the US Centers for Disease Control and Prevention. The study data are freely available for use by researchers from all over the world. We selected cases with serum *T. gondii* antibody test results for the present study. All individuals were followed up until December 2015, and the data on survival status and death dates were collected. The corresponding mortality dataset was obtained from the US National Center for Health Statistics (NCHS), which is also freely accessible. All NHANES protocols had been approved by the NCHS ethics review board, and participants or their proxies had provided informed consent prior to participation. As the present study only utilized anonymized data available to the public, no additional ethics approval was required.

### Definitions

The presence of *T. gondii* antibody was tested using the indirect enzyme immunoassay method. The diagnostic threshold for *T. gondii* infection was 7 IU/ml as described in the original report. Samples with results ≥ 7 IU/ml were considered to indicate *T. gondii* infection. The entire dataset and details on the methods used can be retrieved from the NHANES website (https://www.cdc.gov/nchs/nhanes/about_nhanes.htm).

Hypertension was defined as a systolic blood pressure ≥ 135 mmHg or diastolic blood pressure  ≥ 85 mmHg. Patients with a significant history of hypertension and/or undertreatment of hypertension were also included.

People with type 2 diabetes were defined as having a history of diabetes, currently using insulin or oral hypoglycemic agents, with a fasting blood glucose (FBG)  ≥ 7.0 mmol/l or glycated hemoglobin (HbA1c) ≥ 6.5% or 2-h postprandial glucose  ≥ 11.0 mmol/l.

Body mass index (BMI, kg/m^2^) was defined as the weight in kilograms divided by the square of the height in meters.

Income was measured based on the poverty income ratio, an index reflecting the ratio of household income to the household poverty level determined by area of residence and household size. Low family income was defined as a ratio below 1.

In NHANES III, data on education was collected as a numerical variable (number of years of education), ranging from zero to 17 years. Low educational level was defined as an educational level of lower than the 12th grade.

### Laboratory examinations

Routine laboratory examinations, including blood routine tests, C-reactive protein (CRP), FBG, HbA1c, alanine aminotransferase (ALT), aspartate aminotransferase (AST), albumin, serum, creatinine, blood urea nitrogen (BUN) and uric acid, among others, were all collected from the dataset.

### Statistical analysis

Categorical variables were expressed as percentages. Continuous variables were expressed as means ± standard deviation. The Student t-test (for variables normally distributed), the Mann–Whitney U-test (for variables non-normally distributed) and the Chi-square test (for categorical variables) were used to compare the differences between the seropositive and seronegative groups. We used propensity score matching (PSM) to select age- and sex-matched participants between the two groups. Patients were matched 1:1 based on their propensity scores, and the match tolerance value was 0.0001. The Cox proportional hazards model was used to evaluate the effect of *T. gondii* infection on all-cause mortality. All tests were two-tailed and a* P*-value < 0.05 was considered to be statistically significant. All analysis was conducted in R 3.6.2 (https://www.r-project.org/).

## Results

### Baseline characteristics of the study population

**Fig. 1:**
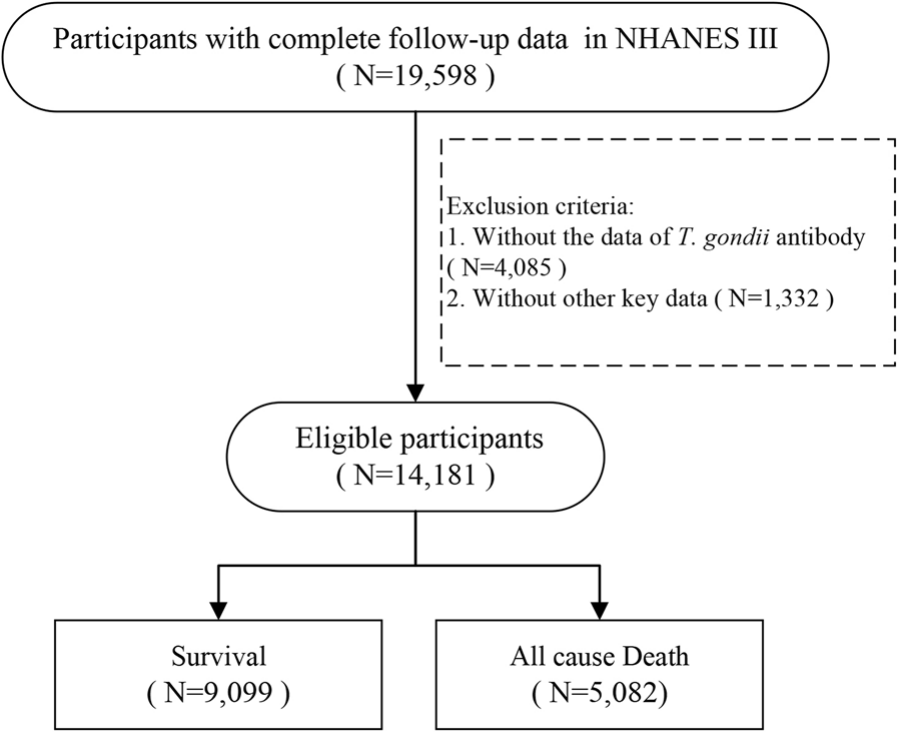
Flow chart of case selection. NHANES, Third National Health and Nutrition Examination Survey

A total of 19,598 participants with follow-up data were screened for this study (Fig. 1). After excluding participants without *T. gondii* antibody test (*N* = 4,085), 1332 without other key data, 14,181 cases were included in final analysis. Among them 6,672 (47.0%) were male and the average age was 46.3 ± 19.5 years old. A total of 2128 (15.0%) had diabetes and 5859 (41.3%) had hypertension. A total of 3831 (27.0%) cases were seropositive in *T. gondii* antibody. More details were shown in Table 1.

**Table 1:** Comparison of *Toxoplasma gondii*-seropositive and -seronegative groups before propensity score matching

Variables	Total	*T. gondii* antibody		*P*
		Negative	Positive	
*N*	14,181	10,350	3831	
All-cause mortality, *n* (%)	5082 (35.8)	3166 (30.6)	1916 (50.0)	< 0.001
Follow-up time (years)	22.5 [16.3, 24.5]	22.8 [19.8, 24.7]	21.7 [10.8, 24.1]	< 0.001
*Race, n (%)*				< 0.001
- Non-Hispanic white	5827 (41.1)	4116 (39.8)	1711 (44.7)	
- Non-Hispanic black	3882 (27.4)	2922 (28.2)	960 (25.1)	
- Mexican–American	3912 (27.6)	2994 (28.9)	918 (24.0)	
- Other	560 (3.9)	318 (3.1)	242 (6.3)	
Male,* n* (%)	6672 (47.0)	4760 (46.0)	1912 (49.9)	< 0.001
Age (years)	46.3 ± 19.5	43.6 ± 18.7	53.7 ± 19.7	< 0.001
Low family income,* n* (%)	3068 (21.6)	2204 (21.3)	864 (22.6)	0.111
Low educational level,* n* (%)	5637 (39.8)	3755 (36.3)	1882 (49.1)	< 0.001
Type 2 diabetes, *n* (%)	2128 (15.0)	1353 (13.1)	775 (20.2)	< 0.001
Hypertension, *n* (%)	5859 (41.3)	3935 (38.0)	1924 (50.2)	< 0.001
BMI (kg/m^2^)	27.1 ± 5.7	26.9 ± 5.8	27.4 ± 5.6	< 0.001
Waist (cm)	92.9 ± 14.6	92.2 ± 14.7	94.9 ± 14.1	< 0.001
White blood cell (× 10^9^/l)	7.2 ± 2.3	7.2 ± 2.3	7.2 ± 2.5	0.567
Hemoglobin (g/l)	139.4 ± 15.2	139.4 ± 15.2	139.3 ± 15.2	0.854
Platelets (× 10^9^/l)	274.1 ± 71.1	275.9 ± 70.7	269.2 ± 71.9	< 0.001
CRP (mg/dl)	0.5 ± 0.8	0.5 ± 0.7	0.5 ± 0.9	0.005
FBG (mmol/l)	5.5 ± 2.1	5.5 ± 2	5.8 ± 2.3	< 0.001
HbA1c (%)	5.5 ± 1.1	5.5 ± 1.1	5.7 ± 1.2	< 0.001
Cholesterol (mmol/l)	5.3 ± 1.2	5.2 ± 1.2	5.4 ± 1.2	< 0.001
Triglyceride (mmol/l)	1.6 ± 1.3	1.6 ± 1.3	1.7 ± 1.4	< 0.001
AST (U/l)	19 [16, 24]	19 [16, 24]	19 [17, 24]	< 0.001
ALT (U/l)	14 [10, 20]	14 [10, 20]	14 [10, 19]	0.046
Albumin (g/l)	41.4 ± 3.8	41.5 ± 3.8	41.1 ± 3.7	< 0.001
Creatinine (μmol/l)	88.4 [79.6, 106.1]	88.4 [79.6, 106.1]	97.2 [79.6, 106.1]	< 0.001
BUN (mmol/l)	5.1 ± 2.0	4.9 ± 1.9	5.5 ± 2.3	< 0.001
Uric acid (μmol/l)	318.1 ± 88.1	314.3 ± 86.8	328.5 ± 90.7	< 0.001

Values in table are presented as the mean ± standard deviation (SD) or as the median with the interquartile range in square brackets, unless indicated otherwise

*ALT* Alanine aminotransferase, *AST* aspartate aminotransferase, *BMI* body mass index, *BUN* blood urea nitrogen, *CRP* C-reactive protein, *FBG* fasting blood glucose, *HbA1c* glycosylated hemoglobin, *BUN* blood urea nitrogen.

### Comparison between the *T. gondii*-seropositive group and the *T. gondii*-seronegative group before and after PSM

According to the results of the *T. gondii* antibody test, participants were divided into a *T. gondii*-seropositive group and a *T. gondii*-seronegative group. The comparison of the baseline characteristics before PSM are shown in Table 1. Patients in the seropositive group were significantly older than their seronegative counterparts (mean ± SD, 53.7 ± 19.7 vs. 43.6 ± 18.7; *P* < 0.001). There was a higher proportion of males in the seropositive group than in the seronegative group (49.9% vs. 46.0%; *P* < 0.001). Diabetes, hypertension and metabolic derangements were more common in the seropositive group.

As age and sex are factors closely associated with life expectancy, and a significant difference in these factors was found between the seropositive and seronegative group, we used PSM to match age and sex between two groups. A total of 3806 pairs of cases were selected in which age and sex were comparable after PSM (Table 2). It was noteworthy that the severity of metabolic derangements, the proportion of patients with diabetes and BMI level remained significantly different between two groups even after matching for age and sex.

**Table 2:** Comparison between the *T. gondii*-seropositive and -seronegative groups after propensity score matching

Variables	Total	*T. gondii* antibody		*P*
		Negative	Positive	
*N*	7612	3806	3806	0.048
All-cause mortality, *n* (%)	3694 (48.5)	1801 (47.3)	1893 (49.7)	0.037
Follow-up time (years)	21.8 [11.4, 24.2]	21.8 [11.8, 24.3]	21.8 [10.9, 24.1]	0.001
*Race, n (%)*				< 0.001
- Non-Hispanic white	3196 (42.0)	1503 (39.5)	1693 (44.5)	
- Non-Hispanic black	2345 (30.8)	1385 (36.4)	960 (25.2)	
- Mexican–American	1735 (22.8)	821 (21.6)	914 (24.0)	
- Other	336 (4.4)	97 (2.5)	239 (6.3)	
Male (%)	3784 (49.7)	1892 (49.7)	1892 (49.7)	1.000
Age (years)	53.5 ± 19.7	53.5 ± 19.7	53.5 ± 19.7	1.000
Low family income,* n* (%)	1673 (22)	812 (21.3)	861 (22.6)	0.184
Low educational level,* n* (%)	3438 (45.2)	1571 (41.3)	1867 (49.1)	< 0.001
Type 2 diabetes, *n* (%)	1453 (19.1)	686 (18.0)	767 (20.2)	0.020
Hypertension, *n* (%)	3764 (49.4)	1858 (48.8)	1906 (50.1)	0.281
BMI (kg/m^2^)	27.2 ± 5.7	26.9 ± 5.7	27.4 ± 5.6	< 0.001
Waist (cm)	94.3 ± 14.2	93.8 ± 14.3	94.9 ± 14.1	< 0.001
White blood cell (×10^9^/l)	7 ± 2.4	6.9 ± 2.3	7.2 ± 2.5	< 0.001
Hemoglobin (g/l)	134.8 ± 15.3	130.3 ± 14.1	139.2 ± 15.1	< 0.001
Platelets (×10^9^/l)	271.7 ± 74	273.9 ± 75.9	269.4 ± 71.9	0.008
CRP (mg/dl)	0.5 ± 0.9	0.5 ± 0.9	0.5 ± 0.9	0.111
FBG (mmol/l)	5.7 ± 2.2	5.6 ± 2.1	5.8 ± 2.2	0.002
HbA1c (%)	5.7 ± 1.2	5.7 ± 1.1	5.7 ± 1.2	0.688
Cholesterol (mmol/l)	5.4 ± 1.2	5.3 ± 1.2	5.4 ± 1.2	< 0.001
Triglyceride (mmol/l)	1.7 ± 1.3	1.6 ± 1.2	1.7 ± 1.4	< 0.001
AST (U/l)	19 [16, 24]	19 [16, 23]	19 [17, 24]	0.002
ALT (U/l)	13 [10, 18]	13 [9, 17]	14 [10, 19]	< 0.001
Albumin (g/l)	40.9 ± 3.9	40.6 ± 4.0	41.1 ± 3.7	< 0.001
Creatinine (μmol/l)	97.2 [79.6, 106.1]	97.2 [79.6, 106.1]	97.2 [79.6, 106.1]	0.224
BUN (mmol/l)	5.4 ± 2.3	5.4 ± 2.3	5.5 ± 2.3	0.453
Uric acid (μmol/l)	323.4 ± 90.5	318.7 ± 90.1	328.2 ± 90.6	< 0.001

Values in table are presented as the mean ± SD or as the median with the interquartile range in square brackets, unless indicated otherwise

### All-cause mortality in *T. gondii*-seropositive and -seronegative groups

**Fig. 2:**
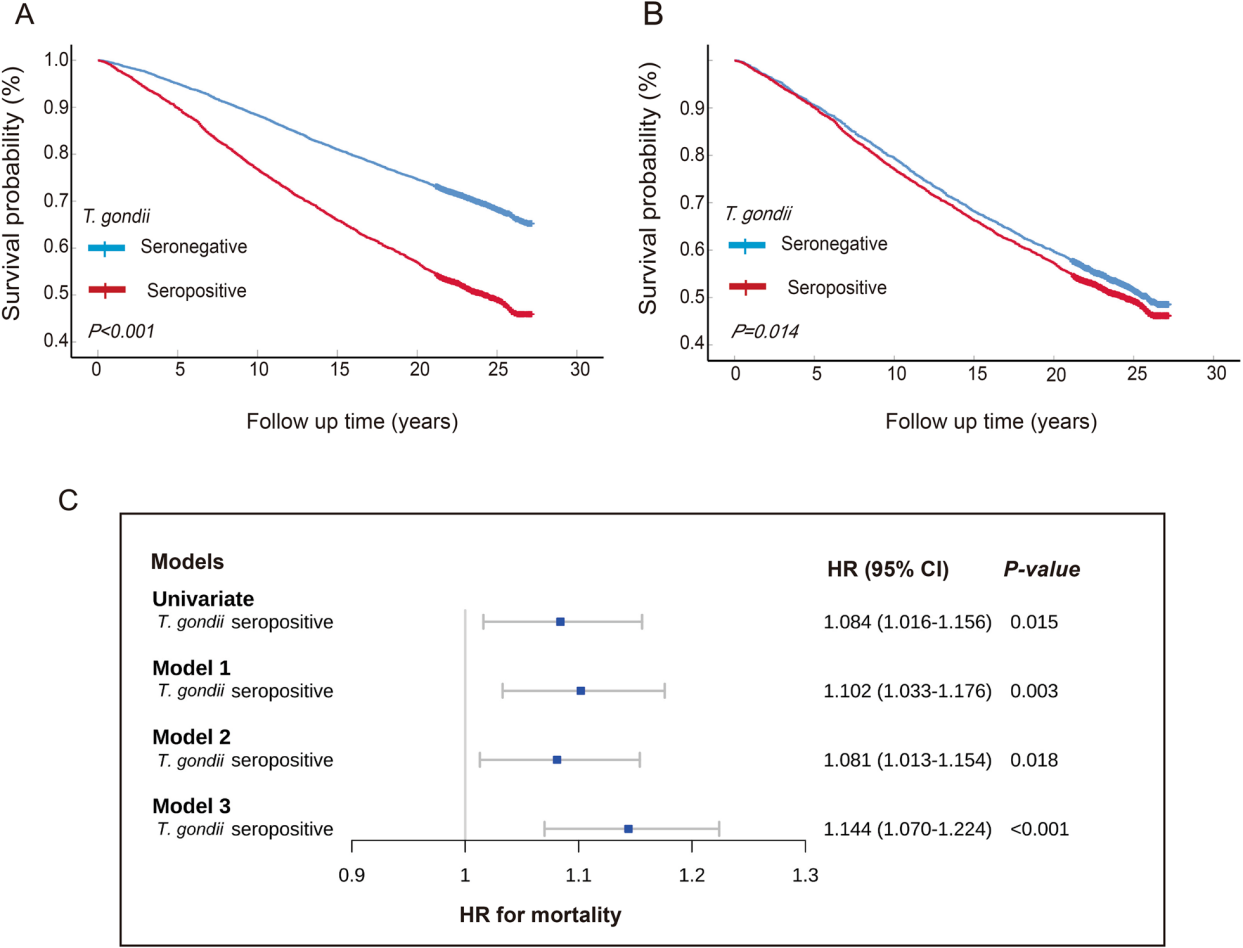
Kaplan–Meier analysis between the *Toxoplasma gondii*-seropositive group and the *T. gondii*-seronegative group before PSM (**a**) and after PSM (**b**). The *P*-values were calculated by the log-rank test. **c** Forest plot of the hazard ratio for mortality based on Cox regression analysis. *CI* Confidence interval, *HR* hazard ratio, *PSM* Propensity score matching

The median follow-up time of the whole cohort was 22.5 (interquartile range 16.3, 24.5) years. A total of 5082 deaths were observed in this cohort, which is a mortality rate of 35.8%. All-cause mortality was significantly higher in the seropositive group than in the seronegative group (50.0% vs. 30.6%; *P* < 0.001). Kaplan–Meier analysis showed a significant difference in the survival time between two groups (Fig. 2a).

After PSM, the difference in the mortality rate between the seropositive group and seronegative group was attenuated but remained statistically significant (49.7% vs. 47.3; *P* = 0.037). The same result was obtained in the Kaplan–Meier analysis (*P* = 0.014; Fig. 2b).

### Cox multivariate analysis for the impact of *T. gondii* infection on all-cause mortality after PSM

We used Cox analysis to explore the correlation between *T. gondii* infection and all-cause mortality in the PSM population. The results are shown in Table 3. In the univariate analysis, *T. gondii* antibody seropositivity, race, sex, age, low educational level, hypertension, diabetes, waist circumference, related metabolic parameters and liver function and renal function test results were significantly different between the survival group and mortality group. Variables with a *P*-value < 0.05 in Table 3 were considered to be candidates for inclusion in the multivariate regression. Collinearity analysis showed a strong collinearity between BMI and waist, FBG and HbA1c, AST and ALT, and creatinine and BUN. For the variables with collinearity, we used the receiver operating characteristic (ROC) analysis to select the variable with a higher area under the ROC curve. The results showed that waist, HbA1c, AST and BUN were the most suitable for inclusion in the multivariate Cox regression analysis.

**Table 3:** Comparison between the survival and all-cause mortality groups after propensity score matching

Variables	Survival	All-cause mortality	*P*
*N*	3918	3694	< 0.001
*T. gondii* seropositive, *n* (%)	1913 (48.8)	1893 (51.2)	0.037
Follow-up time (years)	24.1 [22.5, 25.5]	11 [6.2, 17.0]	< 0.001
*Race,* *n (%)*			< 0.001
- Non-Hispanic white	1207 (30.8)	1989 (53.8)	
- Non-Hispanic black	1423 (36.3)	922 (25.0)	
- Mexican–American	1048 (26.7)	687 (18.6)	
- Other	240 (6.1)	96 (2.6)	
Male, *n* (%))	1810 (46.2)	1974 (53.4)	< 0.001
Age (years)	40.6 ± 14.5	67.2 ± 14.4	< 0.001
Low family income, *n* (%)	896 (22.9)	777 (21.0)	0.057
Low educational level, *n* (%)	1365 (34.8)	2073 (56.1)	< 0.001
Type 2 diabetes, *n* (%)	362 (9.2)	1091 (29.5)	< 0.001
Hypertension, *n* (%)	1216 (31.0)	2548 (69.0)	< 0.001
BMI (kg/m^2^)	27.1 ± 5.8	27.2 ± 5.6	0.819
Waist (cm)	91.7 ± 14.3	97.1 ± 13.6	< 0.001
White blood cell (×10^9^/l)	6.9 ± 2.1	7.2 ± 2.7	< 0.001
Hemoglobin (g/l)	133.7 ± 16.3	136 ± 14.1	< 0.001
Platelets (× 10^9^/l)	278.3 ± 71	264.6 ± 76.4	< 0.001
CRP (mg/dl)	0.4 ± 0.8	0.6 ± 1.0	< 0.001
FBG (mmol/l)	5.3 ± 1.4	6.2 ± 2.7	< 0.001
HbA1c (%)	5.4 ± 0.9	6.0 ± 1.3	< 0.001
Cholesterol (mmol/l)	5.1 ± 1.1	5.6 ± 1.2	< 0.001
Triglyceride (mmol/l)	1.5 ± 1.2	1.8 ± 1.5	< 0.001
AST (U/l)	19 [16, 24]	20 [17, 24]	< 0.001
ALT (U/l)	14 [10, 20]	12 [9, 17]	< 0.001
Albumin (g/l)	41.4 ± 4.0	40.3 ± 3.7	< 0.001
Creatinine (μmol/l)	88.4 [79.6, 106.1]	97.2 [88.4, 114.9]	< 0.001
BUN (mmol/l)	4.7 ± 1.5	6.2 ± 2.7	< 0.001
Uric acid (μmol/l)	306.3 ± 83.0	341.6 ± 94.4	< 0.001

Values in table are presented as the mean ± SD or as the median with the interquartile range in square brackets, unless indicated otherwise

In order to explore the association between *T. gondii* infection and mortality, we established three models with the combination of different variables to adjust for different confounding factors. Model 1 was adjusted for race, sex and age; model 2 was adjusted for race, sex, age, low educational level, diabetes and hypertension; and model 3 was adjusted for additional metabolic profiles and biomarkers of liver and renal functions based on model 2. The results were sustained as being statistically significant in the different models (model 1: hazard ratio [HR] 1.102, 95% confidence interval [CI] 1.033–1.176, *P* = 0.003; model 2: HR 1.081, 95% CI 1.013–1.154, *P* = 0.018; model 3: HR 1.144, 95% CI 1.070–1.224, *P* < 0.001), indicating the association of *T. gondii* infection and all-cause mortality was independent of other confounders (Table 4; Fig. 2c).

**Table 4:** Multivariate COX regression analysis after propensity score matching

Variable	HR	HR (95% CI)	*P*
Univariate analysis			
* T. gondii* seropositive	1.084	1.016–1.156	0.015
Model 1			
* T. gondii* seropositive	1.102	1.033–1.176	0.003
* Race*			
- Non-Hispanic white	1.000		
- Non-Hispanic black	1.156	1.066–1.253	< 0.001
- Mexican–American	1.010	0.925–1.104	0.820
- Other	0.658	0.536–0.808	< 0.001
Male (%)	1.489	1.395–1.589	< 0.001
Age (years)	1.086	1.083–1.089	< 0.001
Model 2			
*T. gondii* seropositive	1.081	1.013–1.154	0.018
* Race*			
- Non-Hispanic white	1.000		
- Non-Hispanic black	1.012	0.931–1.100	0.781
- Mexican–American	0.833	0.758–0.915	< 0.001
- Other	0.620	0.505–0.763	< 0.001
Male (%)	1.493	1.399–1.594	< 0.001
Age (years)	1.080	1.077–1.083	< 0.001
Low educational level	1.325	1.235–1.421	< 0.001
Type 2 diabetes, *n* (%)	1.443	1.343–1.551	< 0.001
Hypertension, *n* (%)	1.256	1.167–1.352	< 0.001
Model 3			
*T. gondii* seropositive	1.144	1.070–1.224	< 0.001
* Race*			
- Non-Hispanic white	1.000		
- Non-Hispanic black	0.920	0.841–1.007	0.070
- Mexican–American	0.816	0.741–0.898	< 0.001
- Other	0.581	0.473–0.715	< 0.001
Male (%)	1.590	1.466–1.724	< 0.001
Age (years)	1.077	1.073–1.080	< 0.001
Low educational level)	1.313	1.224–1.409	< 0.001
Type 2 diabetes, *n* (%)	1.222	1.119–1.335	< 0.001
Hypertension, *n* (%)	1.256	1.165–1.354	< 0.001
Waist (cm)	0.994	0.992–0.997	< 0.001
White blood cell (× 10^9^/l)	1.031	1.022–1.040	< 0.001
Hemoglobin (g/l)	0.992	0.989–0.995	< 0.001
Platelets (× 10^9^/l)	1.000	1.000–1.001	0.760
CRP (mg/dl)	1.047	1.015–1.079	0.003
HbA1c (%)	1.094	1.061–1.129	< 0.001
Cholesterol (mmol/l)	0.991	0.960–1.022	0.554
Triglyceride (mmol/l)	1.003	0.977–1.029	0.813
AST (U/l)	1.005	1.003–1.006	< 0.001
Albumin (g/l)	0.973	0.964–0.982	< 0.001
BUN (mmol/l)	1.042	1.026–1.057	< 0.001
Uric acid (μmol/l)	1.001	1.001–1.002	< 0.001

*CI* Confidence interval, *HR* hazard ratio

### The difference in risk factors for mortality in the *T. gondii-*seropositive and *T. gondii-*seronegative groups

 The results of the Cox multivariate analysis in the *T. gondii-*seropositive and *T. gondii-*seronegative groups are shown in Additional file 1: Table S1. The effect of most factors was similar in the two groups, with the exception of CRP. CRP was significantly associated with the risk of mortality in the *T. gondii*-seropositive group (HR 1.070, 95% CI 1.020–1.121, *P* = 0.005), while no statistical significance was found in the seronegative group (*P* = 0.121).

## Discussion

The present study used data from NHANES III to assess the longitudinal relationship between *T. gondii* infection at baseline and subsequent mortality after adjusting for relevant confounding factors. The main finding of this prospective study is that *T. gondii* antibody positivity was associated with increased all-cause mortality.

Toxoplasmosis is generally regarded to be a threat to immunocompromised patients or those with congenital toxoplasmosis [2, 13]. Several studies have also shown a higher risk for mortality from natural causes in persons with schizophrenia infected with *T. gondii* [4, 11, 12, 14]. Our study is the first to show that *T. gondii* infection is associated with higher mortality in the general population.

Several possible hypotheses might explain this finding. First, *T. gondii* infections are associated with elevated biomarkers of chronic inflammation, such as CRP [15]. Systemic chronic inflammation can lead to several diseases and cause disability and mortality worldwide [16]. The results of the present study show that the CRP level was associated with higher mortality in the *T. gondii*-seropositive group, indicating the possible role of chronic inflammation in an increased mortality. Second, *T. gondii* can influence host metabolism of fatty acids, lipids and energy in the liver [17]. Higher seroprevalence of *T. gondii* antibody has been found in patients with non-alcoholic fatty liver disease [8], type 2 diabetes [9] and cardiovascular disease burden [3]. The higher risk of mortality in the toxoplasmosis population might be partially attributed to the increased metabolic morbidity following *T. gondii* infection. Third, toxoplasmosis has been showed to be linked to some neuropsychiatric disorders [18] and a higher risk of traffic accidents and suicide attempts [19, 20]. The mechanism underlying the impact of toxoplasmosis on emotional and mental problems includes proinflammatory immune response to infection and endocrine and neurotransmitter dysregulation [19].

This study was based on a general population survey database and had a larger sample size and longer follow-up duration than other similar studies. Although the data are strong due to the number and quality of the data, as well as the multiple confounders we have tried to control, there are many possible explanations of this statistically significant association. *Toxoplasma gondii* infection has been shown to be associated with deregulated metabolic functions [21, 22] and cardiovascular disease burden [3]. The factors that increase metabolic diseases can also increase the risk for toxoplasmosis acquisition. We have attempted to control various known confounding factors, including metabolic profiles, socioeconomic status and the medical history, but even with very good multivariate models, using this type of data it is impossible to separate which factor is really influencing the outcome. Notably, the effect of toxoplasmosis on mortality was not as strong as that of diabetes, hypertension and sex. Moreover, as shown in the Fig. 2, the entire effect of toxoplasmosis attenuated when age and sex were controlled. Therefore, we are unable to rule out the possibility that the rest of the effect was mediated by other, yet unknown confounding factor(s). A well-designed prospective study involving people with metabolic risk may possibly answer this question.

## Conclusions

In conclusion, in this epidemiologic follow-up study, toxoplasmosis was found to be associated with higher all-cause mortality in the general population. The results suggest that toxoplasmosis remains a neglected parasitic infection which requires more public health action.

## Supplementary Information

**Additional file 1:** **Table S1**. Multivariate COX regression analysis grouped by the *T. gondii* antibody

## Data Availability

Publicly available datasets were analyzed in this study. The raw data used in the article are available on National Health and Nutrition Examination Survey website (https://www.cdc.gov/nchs/nhanes/index.htm).

## Abbreviations

ALT: Alanine aminotransferase
AST: Aspartate aminotransferase
BMI: Body mass index
BUN: Blood urea nitrogen
CRP: C-reactive protein
FBG: Fasting blood glucose
HbA1c: Glycosylated hemoglobin
HR: Hazard ratio
NHANES III: The Third National Health and Nutrition Examination Survey
PSM: Propensity score matching
