# Supplementary material for: The association between Toxoplasma infection and mortality: the NHANES epidemiologic follow-up study
Source: Parasit Vectors. 2022 Aug 6;15:284. doi: 10.1186/s13071-022-05398-1 (PMC9357320; doi:10.1186/s13071-022-05398-1)
Supplement: Supplementary file 1 — Additional file 1: Table S1. Multivariate COX regression analysis grouped by the T. gondii antibody [file 13071_2022_5398_MOESM1_ESM.doc]

Additional Table S1 Multivariate COX regression analysis grouped by the *T. gondii* antibody

| Variable | *T. gondii* antibody negative | | | *T. gondii* antibody positive | | |
| --- | --- | --- | --- | --- | --- | --- |
|  | HR | HR (95%CI) | *P* | HR | HR (95%CI) | *P* |
| Race |  |  |  |  |  |  |
| Non-Hispanic white | 1.000 |  |  | 1.000 |  |  |
| Non-Hispanic black | 0.928 | 0.816–1.055 | 0.253 | 0.935 | 0.823–1.063 | 0.305 |
| Mexican-American | 0.829 | 0.721–0.954 | 0.009 | 0.819 | 0.716–0.935 | 0.003 |
| Other | 0.412 | 0.276–0.614 | <0.001 | 0.660 | 0.517–0.843 | 0.001 |
| Male (%) | 1.796 | 1.593–2.026 | <0.001 | 1.472 | 1.317–1.645 | <0.001 |
| Age (years) | 1.083 | 1.078–1.088 | <0.001 | 1.071 | 1.067–1.076 | <0.001 |
| Low educational level | 1.249 | 1.127–1.385 | <0.001 | 1.338 | 1.213–1.476 | <0.001 |
| Type 2 diabetes, n (%) | 1.181 | 1.041–1.341 | 0.010 | 1.271 | 1.123–1.438 | <0.001 |
| Hypertension, n (%) | 1.267 | 1.135–1.413 | <0.001 | 1.248 | 1.125–1.385 | <0.001 |
| Waist (cm) | 0.993 | 0.989–0.997 | 0.001 | 0.995 | 0.991–0.999 | 0.014 |
| White blood cell (×10^9^/L) | 1.048 | 1.032–1.065 | <0.001 | 1.024 | 1.012–1.036 | <0.001 |
| Hemoglobin (g/L) | 0.992 | 0.987–0.997 | 0.002 | 0.990 | 0.986–0.994 | <0.001 |
| Platelets (×10^9^/L) | 1.000 | 0.999–1.001 | 0.845 | 1.000 | 0.999–1.001 | 0.879 |
| CRP (mg/dL) | 1.034 | 0.991–1.078 | 0.121 | 1.070 | 1.020–1.121 | 0.005 |
| HbA1c (%) | 1.127 | 1.077–1.179 | <0.001 | 1.064 | 1.018–1.111 | 0.005 |
| Cholesterol (mmol/L) | 1.024 | 0.979–1.071 | 0.306 | 0.968 | 0.926–1.011 | 0.143 |
| Triglyceride (mmol/L) | 0.981 | 0.944–1.020 | 0.346 | 1.021 | 0.988–1.056 | 0.219 |
| AST (U/L) | 1.004 | 1.002–1.006 | <0.001 | 1.006 | 1.003–1.009 | <0.001 |
| Albumin (g/L) | 0.978 | 0.965–0.992 | 0.001 | 0.964 | 0.949–0.978 | <0.001 |
| BUN (mmol/L) | 1.033 | 1.012–1.055 | 0.002 | 1.058 | 1.035–1.082 | <0.001 |
| Uric acid (μmol/L) | 1.001 | 1.000–1.002 | 0.001 | 1.001 | 1.001–1.002 | <0.001 |

**Abbreviations:** *T. gondii: Toxoplasma gondii*; PSM: propensity score matching; BMI: body mass index; HbA1c: glycosylated hemoglobin; AST: aspartate aminotransferase; CRP: C-reactive protein; BUN: blood urea nitrogen; HR: hazard ratio.
